# ZmWRKY83 Negatively Regulates Waterlogging Stress in Maize

**DOI:** 10.3390/plants15152358

**Published:** 2026-07-31

**Authors:** Qiaolu Li, Jingwei Yan, Ya Liu

**Affiliations:** 1 College of Cooperative Economics, Zhejiang Institute of Economics and Trade, Hangzhou 310018, China; liqiaolu@zjiet.edu.cn; 2 College of Forestry (College of Life Sciences), Zhejiang A&F University, Hangzhou 311300, China

**Keywords:** *Zea mays*, ZmWRKY83, waterlogging stress, ZmIAA7, auxin

## Abstract

Waterlogging is a major abiotic stress that restricts maize growth and yield. WRKY transcription factors play important roles in plant stress responses; however, the functions of many maize WRKY members in waterlogging tolerance remain poorly understood. In this study, we investigated the role of ZmWRKY83 in maize responses to waterlogging. *ZmWRKY83* expression was significantly downregulated in maize roots under waterlogging stress. Functional analysis showed that *ZmWRKY83*-overexpressing lines exhibited growth comparable to that of wild-type plants under normal conditions but more severe growth inhibition and lower shoot and root fresh weights after waterlogging treatment. Physiological analyses further revealed that *ZmWRKY83* overexpression impaired adventitious root formation, increased membrane damage, and reduced antioxidant capacity under waterlogging conditions. Transcriptome analysis revealed that the differentially expressed genes were primarily enriched in plant hormone signal transduction, secondary metabolism, and phenylpropanoid biosynthesis. Further investigation identified the auxin-responsive gene *ZmIAA7* as a potential downstream target of ZmWRKY83. qRT-PCR, DLR, and Y1H assays indicated that ZmWRKY83 directly binds to the *ZmIAA7* promoter and represses its transcriptional activity. Collectively, these findings suggest that ZmWRKY83 negatively regulates maize waterlogging tolerance by suppressing auxin-related responses through *ZmIAA7*, expanding the regulatory network underlying WRKY-mediated responses to waterlogging in maize.

## 1. Introduction

Maize is one of the most important crops worldwide and serves as a major source of food, animal feed, and industrial raw materials. Stable maize production is therefore critical for global food security and agricultural development [1]. However, the increasing frequency of extreme rainfall and regional flooding associated with global climate change has made waterlogging a major abiotic stress that limits maize growth and yield formation [1,2]. Maize seedlings are particularly susceptible to waterlogging because their root systems are not fully developed and are highly sensitive to changes in soil aeration and moisture conditions. Prolonged waterlogging restricts oxygen diffusion in the rhizosphere, resulting in hypoxic or anaerobic conditions. These conditions impair root respiration, energy metabolism, nutrient uptake, and ion transport, ultimately causing growth inhibition, leaf chlorosis, reduced root vigor, and even plant death under severe stress [3,4]. Therefore, unraveling the molecular mechanisms underlying maize seedling responses to waterlogging is essential for identifying key tolerance-related genes and improving waterlogging tolerance through maize breeding.

To survive waterlogged conditions, plants have evolved a range of morphological, physiological, and molecular adaptations. The formation of aerenchyma and adventitious roots enhances internal oxygen transport and helps maintain root viability and nutrient and water uptake under oxygen-deficient conditions [5,6,7]. Meanwhile, antioxidant enzymes, including superoxide dismutase (SOD), catalase (CAT), etc., are activated to alleviate oxidative damage caused by excessive reactive oxygen species accumulation [8]. Phytohormones such as ethylene and auxin also participate in waterlogging responses by regulating adventitious root formation, aerenchyma development, stomatal movement, and stress-responsive gene expression [9,10]. Transcription factor families such as ethylene-responsive factors (ERFs), WRKYs, MYBs, and basic helix–loop–helix proteins (bHLHs) function as key regulatory components that connect stress signal perception with downstream gene expression [10,11,12,13].

WRKY transcription factors constitute a large and functionally diverse family in plants [14]. They are characterized by a highly conserved WRKYGQK motif at the N-terminus of the WRKY domain, which mediates sequence-specific DNA binding. WRKY gene families have been systematically identified in numerous plant species, including *Arabidopsis*, soybean, maize, and grape, indicating their evolutionary conservation and functional diversification [15,16]. Functionally, WRKY transcription factors typically modulate downstream gene expression through specifically binding to W-box cis-regulatory elements in target promoters, thereby participating in a wide range of biological processes such as plant growth, developmental regulation, and responses to environmental stresses [17,18,19,20]. Accumulating evidence indicates that WRKY proteins regulate multiple developmental processes, including seed dormancy and germination, trichome formation, plant architecture, root development, and flowering [21,22]. For example, several *Arabidopsis* WRKYs, such as AtWRKY41, regulate seed dormancy and germination by modulating ABA-related genes [21]. In rice and maize, WRKY transcription factors have also been reported to regulate plant height, root development, and other morphological traits through hormone-related pathways involving ABA, auxin, GA, and ethylene signaling [23,24]. In addition to their roles in development, WRKY transcription factors are extensively involved in abiotic stress responses [17]. For example, in *Arabidopsis*, AtWRKY15 participates in salt stress responses [25].

In recent years, increasing attention has been directed toward the roles of WRKY transcription factors in plant responses to waterlogging and hypoxia [26,27]. In kiwifruit, AcWRKY117 and AcWRKY29 directly bind to the promoter of *AcPDC2* and activate its transcription, thereby promoting anaerobic metabolite accumulation and enhancing waterlogging tolerance [26]. In maize, several WRKY transcription factors have also been shown to be implicated in waterlogging responses. For example, ZmWRKY70 enhances waterlogging tolerance by activating hypoxia-responsive genes [28], whereas ZmWRKY122 increases antioxidant capacity and waterlogging tolerance by upregulating the peroxidase gene *ZmPRX101* [29]. Collectively, these findings suggest that maize WRKY transcription factors contribute to waterlogging tolerance by regulating hypoxia adaptation and reactive oxygen species scavenging. Nevertheless, only a limited number of waterlogging-related WRKY genes have been functionally characterized in maize, and most of the characterized genes function as positive regulators [28,29]. It remains unclear whether other WRKY transcription factors exert distinct or opposing regulatory effects and how they directly regulate their downstream target genes. Therefore, identifying additional waterlogging-responsive WRKY transcription factors and elucidating their regulatory mechanisms will advance our understanding of the transcriptional networks governing maize waterlogging tolerance.

In this study, we found that *ZmWRKY83* was significantly downregulated in maize seedlings under waterlogging stress, suggesting a potential role in the waterlogging response. Functional analysis showed that overexpression of *ZmWRKY83* improved the sensitivity of maize seedlings to waterlogging, indicating that ZmWRKY83 negatively regulates waterlogging tolerance. To elucidate the underlying regulatory mechanism, transcriptome analysis and molecular interaction assays were performed to identify potential downstream targets and characterize the role of ZmWRKY83 in maize responses to waterlogging stress. These findings provide new insights into the transcriptional regulation of waterlogging tolerance in maize and identify ZmWRKY83 as a potential genetic target for improving waterlogging-tolerant maize germplasm.

## 2. Results

### 2.1. ZmWRKY83 Negatively Regulates Waterlogging Stress Response in Maize

**Figure 1:**
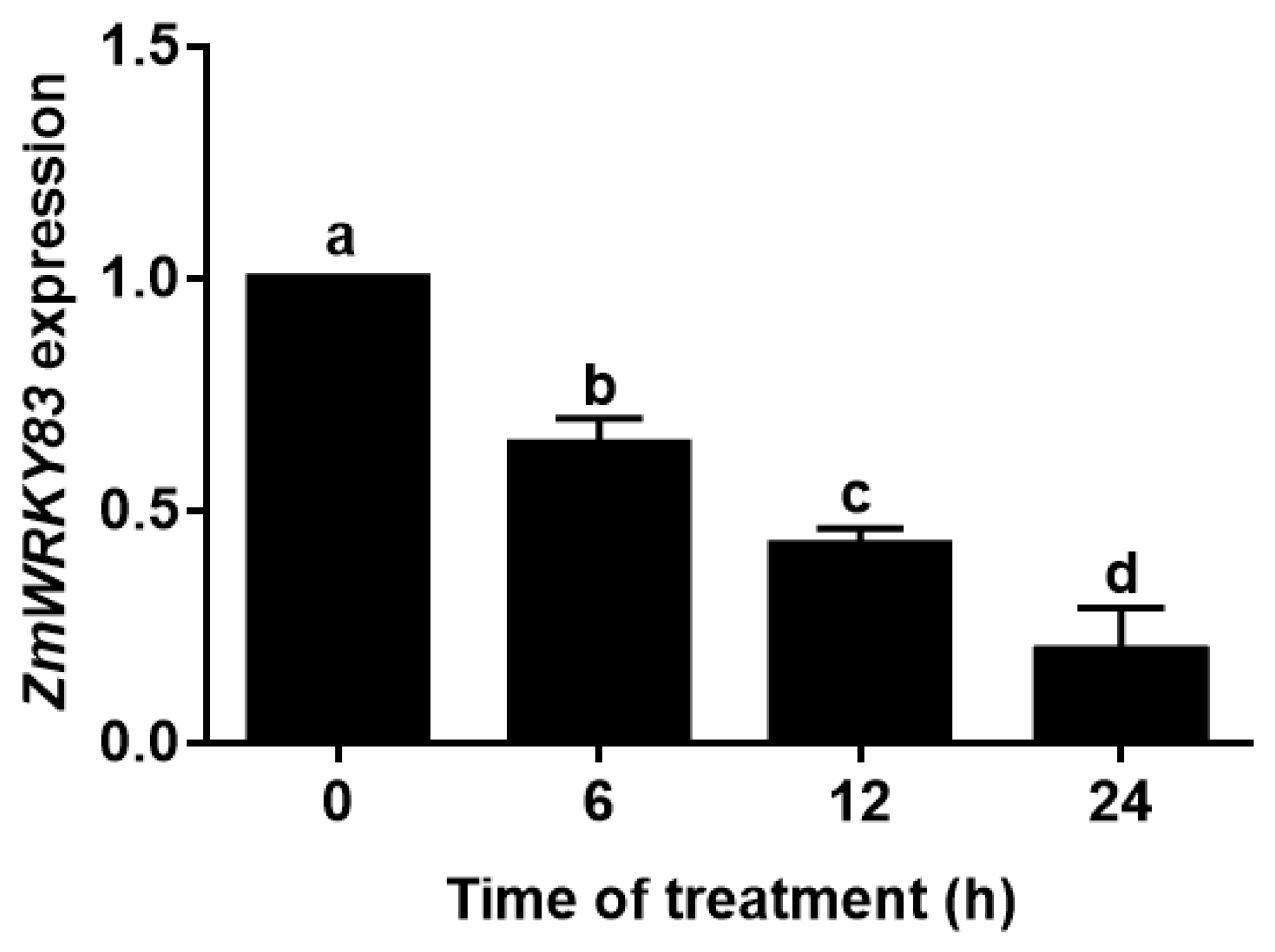
The effect of waterlogging stress on *ZmWRKY83* expression in maize roots. Error bars represent standard deviation (*n* = 3). Different lowercase letters indicate significant differences according to one-way ANOVA (*p* < 0.05).

To determine whether ZmWRKY83 is involved in the response of maize to waterlogging stress, we first analyzed its expression pattern in whole root systems under waterlogging treatment. As shown in Figure 1, the transcript abundance of *ZmWRKY83* progressively decreased with prolonged waterlogging exposure. Compared with the 0 h control, *ZmWRKY83* expression in whole roots was reduced by approximately 35.67%, 57.46%, and 79.21% after 6 h, 12 h, and 24 h of waterlogging treatment, respectively. The lowest expression level was observed at 24 h. These results demonstrate that ZmWRKY83 is highly responsive to waterlogging stress, and its sustained downregulation suggests that it may function as a regulatory component involved in maize adaptation to waterlogging conditions.

**Figure 2:**
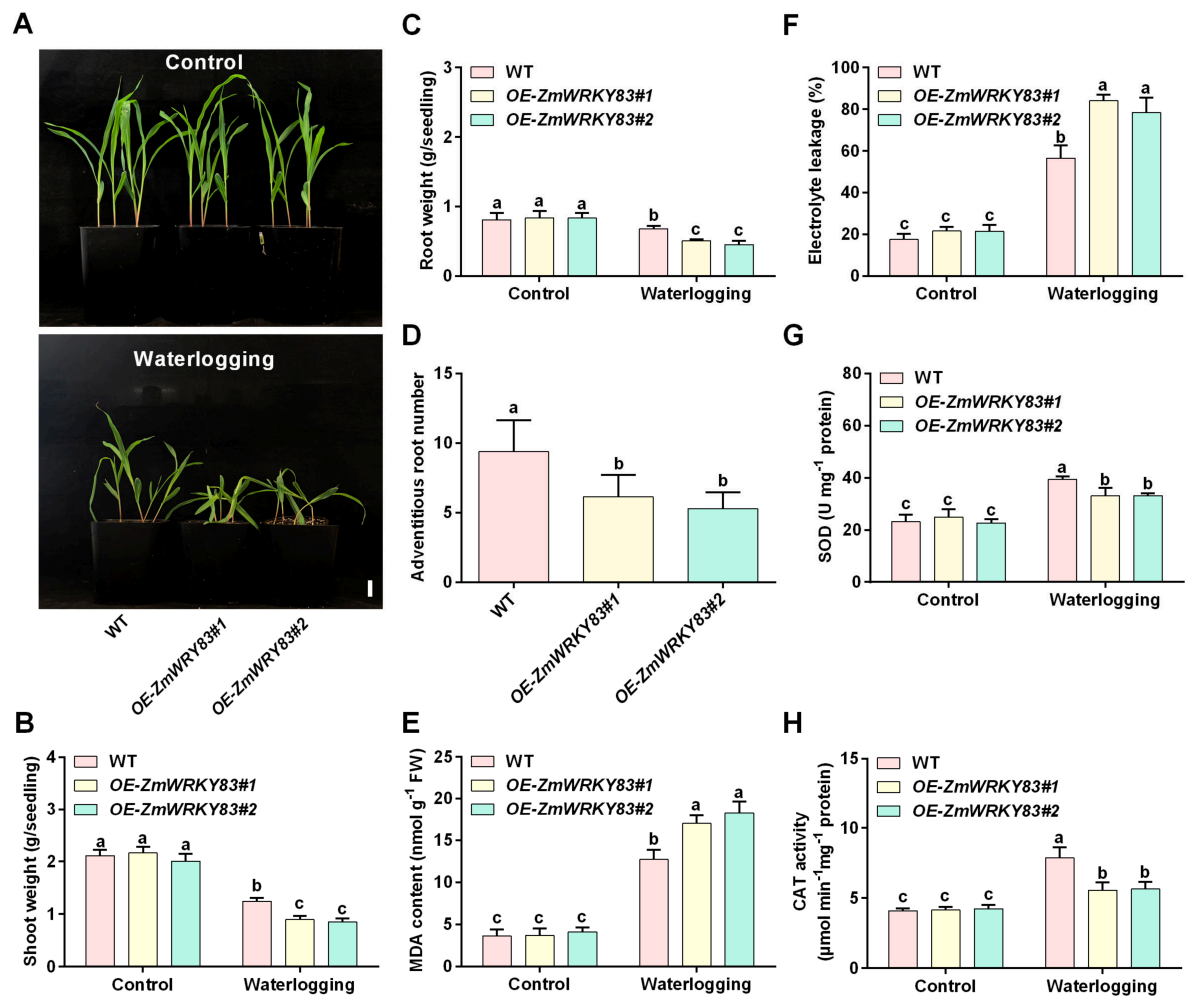
ZmWRKY83 negatively regulates waterlogging tolerance in maize: (**A**) Phenotypic comparison of WT and *ZmWRKY83* overexpression lines under waterlogging conditions. Scale bar = 2.5 cm. (**B**,**C**) Quantitative statistics of shoot and root fresh weights of different genotypes under waterlogging conditions. (**D**) Quantitative statistics of adventitious root number in plants under waterlogging conditions. (**E**–**H**) MDA content (**E**), electrolyte leakage (**F**), SOD activity (**G**), and CAT activity (**H**) in roots of different genotypes under waterlogging conditions. Error bars represent standard deviation (*n* = 3). Different lowercase letters indicate significant differences according to one-way (**D**) or two-way (**B**,**C**,**E**–**H**) ANOVA (*p* < 0.05).

To further investigate the biological function of ZmWRKY83 in maize, we generated *ZmWRKY83*-overexpressing transgenic lines and used them for functional analysis. qRT-PCR analysis confirmed that *ZmWRKY83* transcript levels were significantly increased in two independent overexpression lines, *OE-ZmWRKY83#1* and *OE-ZmWRKY83#2*, compared with wild-type plants. Under waterlogging stress, endogenous *ZmWRKY83* expression was significantly reduced in wild-type plants, whereas *ZmWRKY83* transcript levels remained relatively stable in the overexpression lines, showing no significant change compared with their respective control conditions (Figure S1). Ten-day-old maize seedlings were subjected to waterlogging stress for 6 days. Under normal growth conditions, no obvious phenotypic differences were observed between the *ZmWRKY83* overexpression lines and WT plants. However, following waterlogging treatment, the *ZmWRKY83*-overexpressing lines displayed more pronounced growth inhibition than WT plants, indicating reduced waterlogging tolerance (Figure 2A). Consistently, biomass analysis revealed that waterlogging stress significantly reduced both shoot and root fresh weights in all genotypes. Compared with WT plants, the *ZmWRKY83*-overexpressing lines exhibited greater reductions in shoot and root fresh weights under waterlogging conditions. Specifically, although waterlogging stress reduced shoot and root fresh weights in all genotypes, *OE-ZmWRKY83#1* and *OE-ZmWRKY83#2* plants accumulated significantly less shoot fresh weight than WT plants, with reductions of approximately 28.15% and 31.37%, respectively. Similarly, root fresh weight accumulation was lower by approximately 25.49% and 33.33% in *OE-ZmWRKY83#1* and *OE-ZmWRKY83#2* lines, respectively, compared with WT plants (Figure 2B,C).

Further, under waterlogging stress, the number of adventitious roots was significantly reduced in *ZmWRKY83*-overexpressing lines compared with wild-type plants (Figure 2D). In addition, *OE-ZmWRKY83* plants exhibited higher MDA accumulation than WT plants under waterlogging conditions, indicating enhanced membrane lipid peroxidation (Figure 2E). Waterlogging markedly increased electrolyte leakage in all genotypes, with *OE-ZmWRKY83* lines showing significantly higher leakage than WT plants (Figure 2F). Meanwhile, antioxidant enzyme activities were altered by *ZmWRKY83* overexpression. Compared with WT plants, *OE-ZmWRKY83* lines displayed lower SOD and CAT activities under waterlogging stress (Figure 2G,H), suggesting weakened antioxidant capacity and increased oxidative damage. Two-way ANOVA revealed significant Genotype × Treatment interactions for both shoot fresh weight (F = 6.218, *p* = 0.014), root fresh weight (F = 5.383, *p* = 0.021), MDA content (F = 11.780, *p* = 0.001), electrolyte leakage (F = 11.484, *p* = 0.002), SOD activity (F = 4.975, *p* = 0.027), and CAT activity (F = 13.244, *p* = 0.001), indicating that WT and *ZmWRKY83*-overexpressing plants exhibited differential biomass responses to waterlogging stress. Together, these results suggest that elevated expression of *ZmWRKY83* negatively affects maize performance under waterlogging stress, likely by impairing root adaptation, increasing oxidative damage, and reducing antioxidant capacity.

### 2.2. RNA-Seq Quality Assessment and Global Transcriptional Profiling of WT and ZmWRKY83-Overexpressing Maize Seedlings

**Figure 3:**
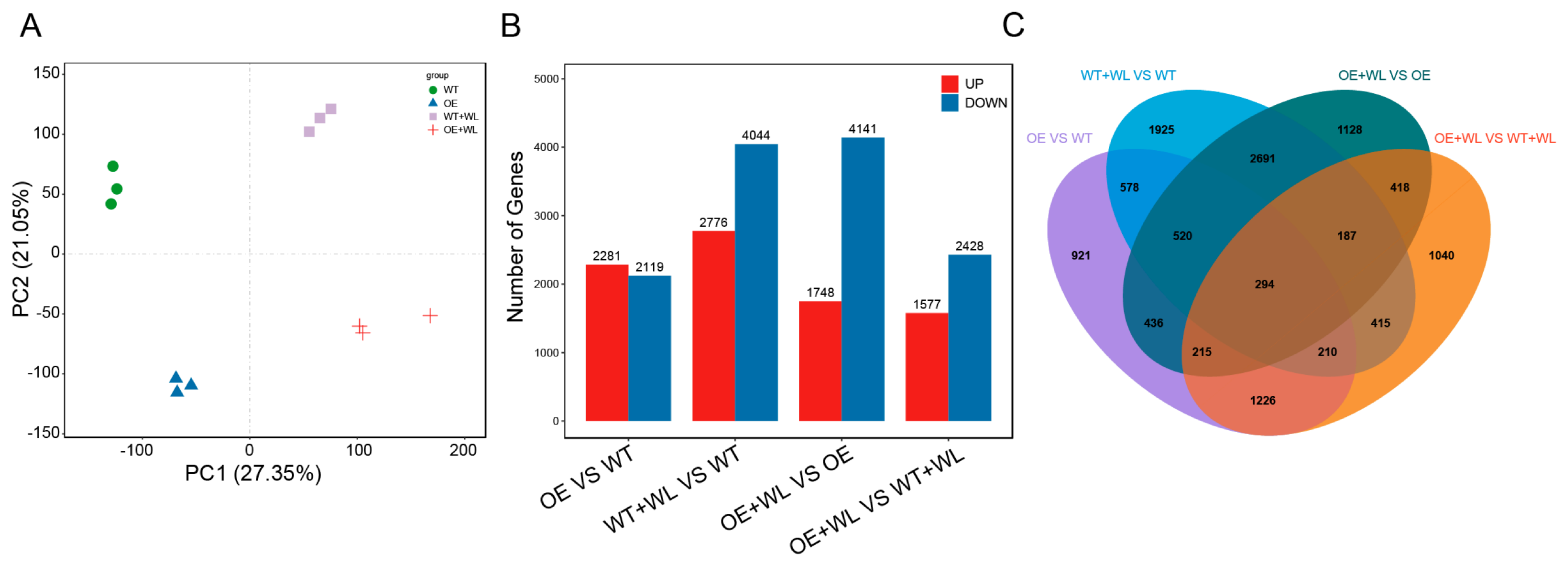
Transcriptome overview of WT and *ZmWRKY83*-overexpressing maize seedlings under waterlogging stress: (**A**) PCA of RNA-seq samples from WT, OE, WT+WL, and OE+WL groups. Each point represents one biological replicate. (**B**) Numbers of upregulated (UP) and downregulated (DOWN) DEGs in distinct comparisons, including OE vs. WT, WT+WL vs. WT, OE+WL vs. OE, and OE+WL vs. WT+WL. (**C**) Venn diagram showing the overlap of DEGs among different comparisons. WT, wild type; OE, *ZmWRKY83*-overexpressing plants; WL, waterlogging treatment.

To investigate the transcriptional changes associated with ZmWRKY83-mediated waterlogging responses, RNA-seq analysis was performed using wild-type (WT) and *ZmWRKY83*-overexpressing maize seedlings (OE) under normal and waterlogging conditions (WL). Four treatment groups were included: WT, wild-type plants under normal conditions; WT+WL, wild-type plants subjected to waterlogging stress; OE, *ZmWRKY83*-overexpressing plants under normal conditions; and OE+WL, *ZmWRKY83*-overexpressing plants subjected to waterlogging stress. The sequencing quality metrics are summarized in Table S2. A total of 44,698,286–52,386,884 clean reads were obtained for each sample, corresponding to 6.73–7.86 Gb of clean bases. The sequencing error rate was low, ranging from 0.02% to 0.03%. The Q20 and Q30 values were 97.22–98.14% and 92.68–94.85%, respectively, indicating high sequencing accuracy. The GC content ranged from 52.89% to 55.29%, with only minor variation among samples. Collectively, these results suggest that the RNA-seq data were of good quality and suitable for subsequent transcriptome analysis. PCA showed that biological replicates within each treatment clustered closely, supporting the reliability of the RNA-seq dataset. The separation of WT, OE, WT+WL, and OE+WL samples along the first two principal components suggested that both waterlogging treatment and genotype contributed to the overall transcriptional variation (Figure 3A). However, PCA provides an overall visualization of transcriptomic variation and does not directly identify the specific biological processes or individual genes responsible for the observed separation among samples.

As shown in Figure 3B, a total of 4400, 6820, 5889, and 4005 DEGs were identified in the comparisons of OE vs. WT, WT+WL vs. WT, OE+WL vs. OE, and OE+WL vs. WT+WL, respectively. Among these comparisons, waterlogging treatment caused extensive transcriptional reprogramming, with 2776 upregulated and 4044 downregulated genes in WT plants and 1748 upregulated and 4141 downregulated genes in OE plants, indicating that waterlogging predominantly induced transcriptional repression. In addition, comparison between OE and WT plants under control conditions identified 2281 upregulated and 2119 downregulated genes, suggesting that overexpression of *ZmWRKY83* altered the basal transcriptional profile. Under waterlogging conditions, OE plants exhibited 1577 upregulated and 2428 downregulated genes compared with WT plants, indicating that *ZmWRKY83* overexpression modified the transcriptional response to waterlogging stress. Venn analysis further revealed the relationships among DEGs from different comparisons (Figure 3C). Among the DEGs induced by waterlogging, 3692 genes were commonly regulated between WT+WL vs. WT and OE+WL vs. OE, suggesting a core waterlogging-responsive gene set independent of *ZmWRKY83* overexpression. Meanwhile, 481 genes were shared between OE+WL vs. WT+WL and the waterlogging-responsive comparisons, indicating that these genes may represent ZmWRKY83-associated responses under waterlogging conditions. These results indicate that ZmWRKY83 reshapes the transcriptomic landscape of maize responses to waterlogging stress.

### 2.3. Transcriptome Analysis Reveals Altered Waterlogging-Responsive Gene Expression in ZmWRKY83-Overexpressing Maize

**Figure 4:**
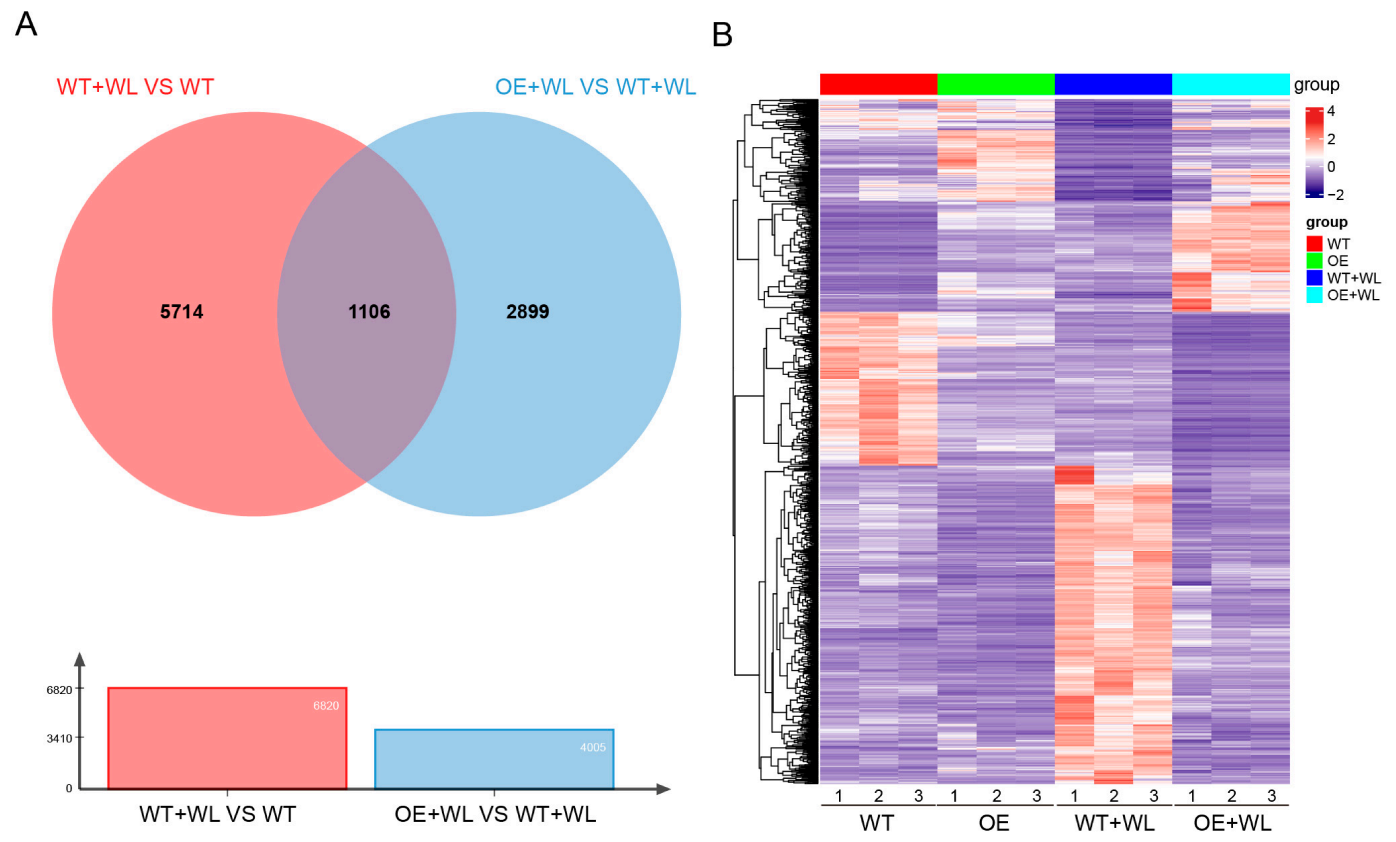
Comparative analysis of differentially expressed genes under waterlogging stress: (**A**) Venn diagram showing the overlap of DEGs between the WT+WL vs. WT and OE+WL vs. WT+WL comparisons. The bar plot below shows the total number of DEGs in each comparison. (**B**) Hierarchical clustering heatmap of DEGs among WT, OE, WT+WL, and OE+WL samples. Gene expression levels were normalized using Z-score transformation based on FPKM values before hierarchical clustering.

To investigate how ZmWRKY83 affects transcriptional responses under waterlogging conditions, differentially expressed genes were examined in the WT+WL versus WT and OE+WL versus WT+WL comparisons. A total of 6820 DEGs were identified in WT plants following waterlogging treatment, indicating substantial transcriptional reprogramming in response to waterlogging stress. In the OE+WL vs. WT+WL comparison, 4005 DEGs were identified, suggesting that *ZmWRKY83* overexpression markedly reshaped gene expression under waterlogging conditions. Venn analysis showed that 1106 DEGs were shared between the two comparisons, whereas 5714 and 2899 DEGs were specific to WT+WL vs. WT and OE+WL vs. WT+WL, respectively (Figure 4A). The 1106 shared DEGs likely represent conserved components of the maize waterlogging response, whereas the genotype-specific DEGs reflect transcriptional changes associated with altered ZmWRKY83 expression. In addition, hierarchical clustering analysis revealed distinct expression patterns among WT, OE, WT+WL, and OE+WL samples, with good consistency among biological replicates (Figure 4B and Table S3). Notably, OE+WL samples displayed distinct expression profiles from WT+WL samples, indicating that *ZmWRKY83* overexpression reshapes the transcriptional landscape under waterlogging conditions.

### 2.4. Functional Enrichment Analysis of Differentially Expressed Genes Under Waterlogging Stress

**Figure 5:**
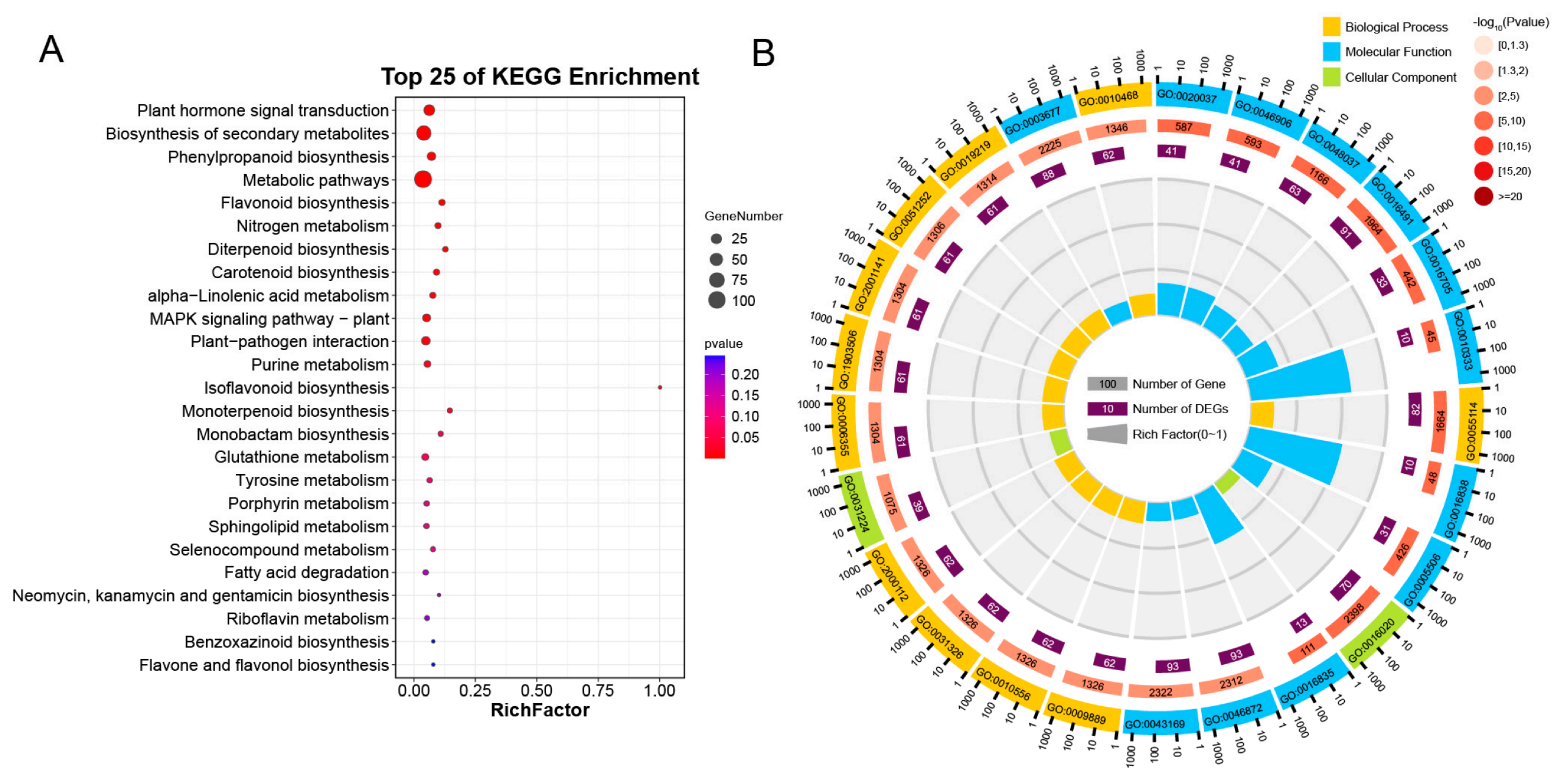
Functional enrichment analysis of DEGs: (**A**) KEGG enrichment analysis of DEGs. The top 25 enriched pathways are shown. (**B**) GO enrichment analysis of DEGs. The circular plot displays gene number, DEG number, and enrichment level for each GO term.

To explore the potential biological functions of the DEGs, KEGG and GO enrichment analyses were conducted. KEGG enrichment analysis identified several significantly enriched pathways, including plant hormone signal transduction, biosynthesis of secondary metabolites, etc. Additionally, several stress-associated pathways, including nitrogen metabolism, carotenoid biosynthesis, etc., were also enriched (Figure 5A). These results suggest that hormone signaling, secondary metabolism, and stress-responsive pathways may contribute to ZmWRKY83-mediated regulation of waterlogging responses. GO enrichment analysis revealed that the DEGs were primarily associated with metabolic processes, cellular activities, stress responses, and regulatory functions (Figure 5B). Together, these enrichment results indicate that ZmWRKY83 may affect maize waterlogging responses by regulating multiple downstream biological processes and metabolic pathways.

### 2.5. ZmWRKY83 Directly Represses the Expression of the Auxin-Responsive Gene ZmIAA7 Under Waterlogging Stress

KEGG enrichment analysis showed that the plant hormone signal transduction pathway was significantly enriched among the differentially expressed genes, suggesting a potential role of hormone-related signaling in ZmWRKY83-regulated waterlogging responses. Considering the important role of auxin signaling in plant adaptation to waterlogging, especially in root development and stress responses [6,30], we further focused on auxin-related DEGs within the plant hormone signal transduction pathway. The candidate genes were selected based on three criteria: (i) significant differential expression between WT+WL and OE+WL plants, (ii) involvement in auxin-related signaling or response, and (iii) potential relevance to root developmental regulation under waterlogging conditions. Based on this screening workflow, *ZmIAA7*, an auxin-responsive Aux/IAA family gene, was identified as a candidate downstream gene associated with ZmWRKY83 regulation.

**Figure 6:**
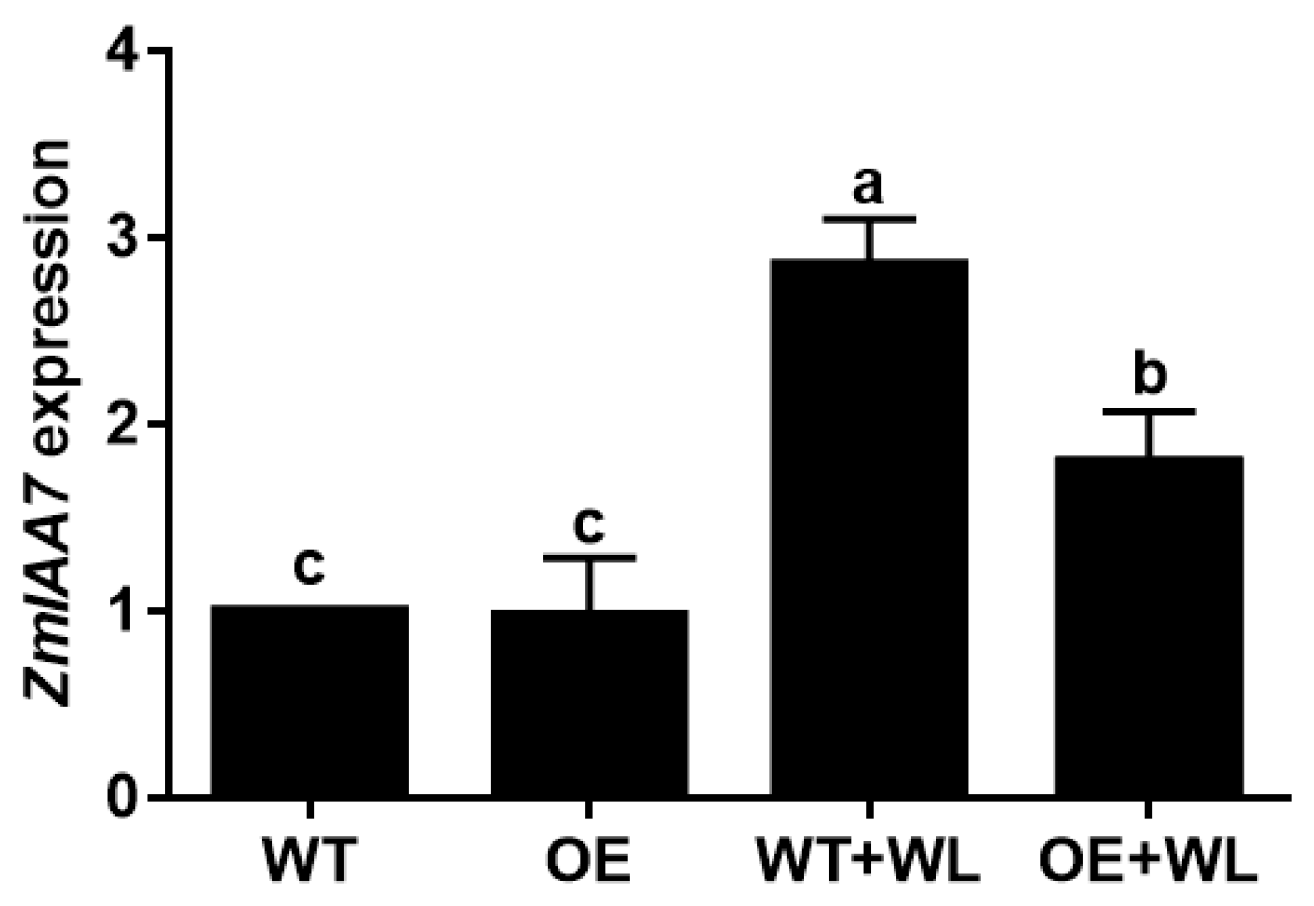
Expression analysis of *ZmIAA7* in WT and *ZmWRKY83*-overexpressing maize seedlings. qRT-PCR analysis of *ZmIAA7* transcript abundance in WT, OE, WT+WL, and OE+WL seedlings. Maize seedlings were subjected to waterlogging stress, and whole root samples were collected for expression analysis. Data are shown as the mean ± SD. Different lowercase letters indicate significant differences among treatments (*p* < 0.05).

To verify the expression pattern of *ZmIAA7*, qRT-PCR analysis was performed in WT and *ZmWRKY83*-overexpressing maize seedlings (OE) under normal and waterlogging conditions. No significant difference in *ZmIAA7* transcript abundance was observed between WT and OE plants under normal growth conditions. However, waterlogging stress significantly upregulated *ZmIAA7* expression in WT plants. Compared with WT+WL plants, the expression level of *ZmIAA7* was significantly lower in OE+WL plants, indicating that *ZmWRKY83* overexpression suppressed the waterlogging-induced *ZmIAA7* expression (Figure 6). These results suggest that ZmIAA7 may be negatively regulated by ZmWRKY83.

**Figure 7:**
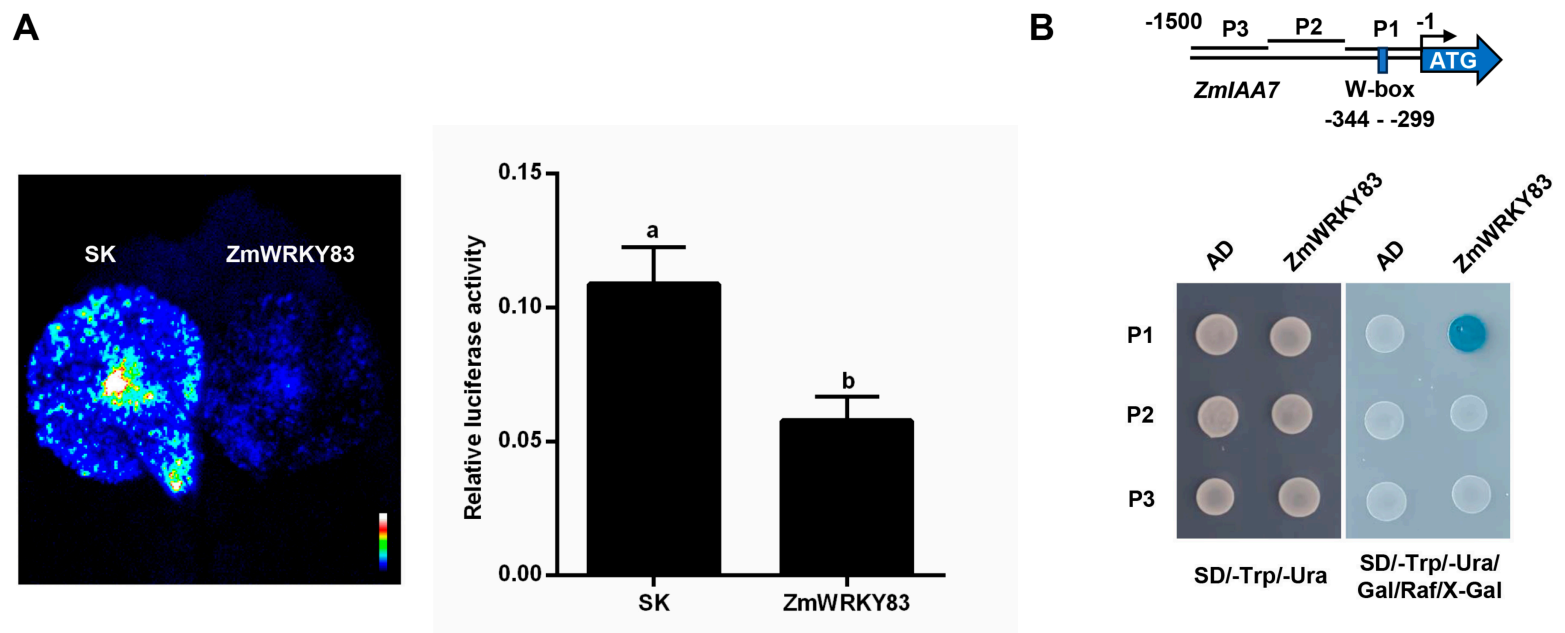
ZmWRKY83 represses the transcriptional activity of the *ZmIAA7* promoter: (**A**) DLR analysis showing the effect of ZmWRKY83 on *ZmIAA7* promoter-driven transcriptional activity in *Nicotiana benthamiana* leaves. The LUC/REN ratio was calculated to represent relative promoter activity. Data are shown as the mean ± SD (*n* = 3), and different lowercase letters indicate significant differences (*p* < 0.05). The color bar denotes signal intensity gradient. (**B**) Yeast one-hybrid assay showing the binding of ZmWRKY83 to different regions of the *ZmIAA7* promoter. The P1, P2, and P3 fragments indicate distinct promoter regions of *ZmIAA7*. The ATG arrow indicates the translation start codon, with −1 representing its upstream position.

To further investigate whether ZmWRKY83 directly regulates *ZmIAA7*, we analyzed the 1500 bp promoter sequence upstream of the *ZmIAA7* translation initiation site. One putative WRKY-binding W-box element (CTGACC) was identified within this promoter region, located from −344 bp to −299 bp upstream of the ATG start codon. A dual-luciferase reporter assay was performed in *Nicotiana benthamiana* leaves. The luminescence imaging showed that co-expression of *ZmWRKY83* reduced the reporter signal driven by the *ZmIAA7* promoter compared with the empty vector control. Consistently, quantitative analysis revealed a significant decrease in relative luciferase activity upon ZmWRKY83 expression, indicating that ZmWRKY83 represses *ZmIAA7* promoter activity (Figure 7A). In addition, yeast one-hybrid assays were conducted using different fragments of the *ZmIAA7* promoter. Yeast cells co-transformed with ZmWRKY83 and the P1 promoter fragment grew normally and showed blue coloration on SD/-Trp/-Ura/Gal/Raf/X-Gal medium, whereas no obvious blue signal was observed for the P2 and P3 fragments. Consistent with the promoter analysis, the P1 fragment contained the W-box sequence (CTGACC), while P2 and P3 lacked this predicted WRKY-binding motif, supporting the sequence-specific interaction between ZmWRKY83 and the *ZmIAA7* promoter. These results indicate that ZmWRKY83 can directly bind to the P1 region of the *ZmIAA7* promoter (Figure 7B). Together, these findings demonstrate that ZmWRKY83 represses *ZmIAA7* transcription by binding to its promoter, thereby negatively regulating the auxin-responsive gene *ZmIAA7* under waterlogging stress.

## 3. Discussion

Maize is affected by waterlogging because hypoxic soil conditions disrupt root respiration, nutrient uptake, and normal seedling development, ultimately leading to yield loss [2]. Thus, elucidating the regulatory mechanisms involved in maize waterlogging responses and discovering key genes associated with tolerance are essential for developing waterlogging-resilient maize varieties through molecular breeding. Although several waterlogging-responsive genes have been reported in maize [29,31], the transcription factors that coordinate this process and their downstream regulatory networks remain largely unexplored. In this study, we characterized the biological function and regulatory mechanism of ZmWRKY83 in maize responses to waterlogging stress. By integrating expression analysis, transgenic phenotyping, transcriptome profiling, and downstream target validation, our results unravel a negative regulatory role of ZmWRKY83 in maize waterlogging tolerance.

WRKY transcription factors are important components of plant stress-response networks, and their expression is often regulated by environmental cues [32,33]. Previous studies on maize WRKY genes involved in waterlogging responses have mainly focused on positive regulators [28,29]. For example, ZmWRKY70 enhances flooding tolerance by activating hypoxia-responsive genes [28], whereas ZmWRKY122 improves waterlogging tolerance by inducing the peroxidase gene *ZmPRX101* and strengthening antioxidant defense [29]. In contrast, our results reveal a negative regulatory role of ZmWRKY83 in maize waterlogging tolerance, highlighting the functional diversification of maize WRKY transcription factors during stress adaptation. Such functional divergence likely arises from the extensive diversification of the maize WRKY family, allowing different members to perform distinct regulatory functions under stress conditions [34,35]. The expression of *ZmWRKY83* gradually decreased in maize roots under waterlogging stress, implying that repression of *ZmWRKY83* may be part of the adaptive response to oxygen-deficient conditions. Consistently, *ZmWRKY83*-overexpressing plants showed no obvious growth difference from WT plants under normal conditions but exhibited more severe growth inhibition and lower shoot and root fresh weights under waterlogging stress. Further physiological analyses revealed that *OE-ZmWRKY83* lines produced fewer adventitious roots, accumulated higher levels of MDA, and showed increased electrolyte leakage under waterlogging conditions, indicating impaired root adaptation and enhanced membrane damage. In addition, *ZmWRKY83* overexpression reduced SOD and CAT activities, suggesting weakened antioxidant capacity and elevated oxidative stress (Figure 2). Together, these findings indicate that sustained high expression of ZmWRKY83 compromises maize waterlogging tolerance by affecting root development and membrane stability. In addition, consistent with the stress-responsive expression pattern of *ZmWRKY83*, analysis of the *ZmWRKY83* promoter uncovered a variety of regulatory elements potentially involved in stress responses, hormone regulation, and transcriptional activity. These findings suggest that *ZmWRKY83* expression may be integrated into complex signaling networks during waterlogging adaptation. These elements, including stress- and hormone-responsive motifs, suggest that ZmWRKY83 expression may be regulated by complex environmental and signaling networks during waterlogging adaptation (Figure S2). Although the functions of individual cis-elements require further validation, these findings support the potential involvement of ZmWRKY83 in stress- and hormone-related regulatory processes.

Transcriptome analysis further supported the involvement of ZmWRKY83 in waterlogging-responsive transcriptional regulation. Compared with WT+WL plants, OE+WL plants displayed 4005 DEGs, indicating that *ZmWRKY83* overexpression substantially reshaped the transcriptional response under waterlogging conditions (Figure 3). Previous studies have shown that waterlogging and hypoxia trigger extensive transcriptional reprogramming in plants, particularly affecting genes associated with energy metabolism, hormone signaling, ROS homeostasis, and stress-defense pathways [8,36]. Consistent with these findings, the DEGs identified in this study were primarily enriched in plant hormone signal transduction and phenylpropanoid pathways, among others. Plant hormones, including ethylene and auxin, are key regulators of plant growth modulation and environmental adaptation, adventitious root formation, aerenchyma development, and stress-defense responses under waterlogging stress [6,7]. For example, in maize, the group VII ethylene-responsive factor ZmEREB180 regulates adventitious root formation under waterlogging conditions by modulating auxin and ethylene biosynthesis and also enhances waterlogging tolerance [37]. In *Arabidopsis*, waterlogging stress induces *RbohD* expression, which regulates H_2_O_2_ accumulation and thereby contributes to the response to waterlogging stress [38]. In cucumber, CsMYB6 directly activates *CsACO2* and *CsGA20ox2* expression, increasing ethylene and gibberellin accumulation and thereby promoting adventitious root formation under waterlogging conditions [39]. In addition, phenylpropanoid and flavonoid pathways are closely associated with antioxidant defense and cell wall remodeling [40,41], while MAPK signaling functions as an important module for stress signal transduction [42,43]. Therefore, ZmWRKY83 may influence maize waterlogging tolerance by regulating hormone-mediated signaling and stress-associated metabolic processes.

Among these enriched pathways, auxin signaling was of particular relevance to the ZmWRKY83-mediated response. Auxin is known to regulate root system remodeling, adventitious root formation, and plant adaptation to oxygen-deficient environments [6,44]. In this study, the auxin-responsive gene ZmIAA7 was identified as a direct target negatively regulated by ZmWRKY83. Because Aux/IAA proteins are key repressors in auxin signaling and are involved in coordinating auxin-mediated growth and stress responses [45,46], altered *ZmIAA7* expression may affect root developmental plasticity under waterlogging conditions. The reduced induction of *ZmIAA7* in *ZmWRKY83*-overexpressing plants suggests that persistent *ZmWRKY83* expression may disrupt auxin-mediated adaptive responses, thereby increasing the sensitivity of maize seedlings to waterlogging stress. Thus, the ZmWRKY83-ZmIAA7 module may represent a potential regulatory connection between WRKY-mediated transcriptional control and auxin signaling during maize waterlogging responses. Interestingly, *ZmWRKY83* overexpression did not significantly affect *ZmIAA7* expression under normal growth conditions. This discrepancy suggests that the regulatory activity of ZmWRKY83 on *ZmIAA7* may be context-dependent and requires additional waterlogging-associated signals. Under normal conditions, *ZmIAA7* expression may be maintained by auxin homeostasis and other transcriptional regulators, which could buffer the effect of elevated ZmWRKY83 abundance. Alternatively, waterlogging stress may activate additional regulatory components, such as stress-induced post-translational modifications or interacting cofactors, that enhance the transcriptional repression activity of ZmWRKY83 toward ZmIAA7. Therefore, the absence of *ZmIAA7* repression under normal conditions does not exclude a regulatory relationship between ZmWRKY83 and ZmIAA7 during waterlogging stress but rather suggests that ZmWRKY83-mediated regulation may depend on the specific stress context.

However, although our Y1H and DLR demonstrate that ZmWRKY83 binds to and represses the *ZmIAA7* promoter, these results alone do not establish that ZmIAA7 is the essential downstream mediator of ZmWRKY83 function in vivo. Moreover, the independent contribution of ZmIAA7 to maize waterlogging tolerance remains to be experimentally validated. Therefore, whether altered *ZmIAA7* expression is sufficient to modulate waterlogging tolerance and whether ZmIAA7 genetically acts downstream of ZmWRKY83 require further investigation. Future studies should further validate the biological function of ZmIAA7 in maize waterlogging tolerance. *ZmIAA7* overexpression and loss-of-function materials could be generated to determine whether altered ZmIAA7 expression affects root growth, auxin-related responses, and waterlogging tolerance. In addition, genetic interaction analysis between ZmWRKY83 and ZmIAA7, such as double transgenic or mutant materials, or complementation assays by introducing ZmIAA7 into *ZmWRKY83*-overexpressing backgrounds, would help clarify whether ZmIAA7 functions as a downstream component of the ZmWRKY83-mediated regulatory pathway in vivo.

## 4. Materials and Methods

### 4.1. Plant Materials and Treatments

*ZmWRKY83*-overexpressing transgenic maize lines were generated in B73 through *Agrobacterium*-mediated genetic transformation [47]. For phenotypic and stress-response analyses, maize seeds were germinated on moist filter paper; then, uniform seedlings were transplanted into pots containing the mixed substrate. Ten-day-old seedlings were subjected to waterlogging treatment for 6 days, with the water level maintained 2 cm above the soil surface.

### 4.2. Phenotypic and Physiological Index Evaluation

Ten-day-old wild-type maize seedlings (WT) and *ZmWRKY83*-overexpressing lines (OE) with similar growth status were subjected to waterlogging stress for 6 days [29]. Following the treatment, fresh weights were determined to assess the growth performance of each genotype under waterlogging conditions. The concentrations of malondialdehyde, superoxide dismutase, and catalase in roots, as well as the amount of electrolyte leakage, were measured as described by [29].

### 4.3. qRT-PCR Analysis

Total RNA was extracted from maize roots using TRIzol reagent and reverse-transcribed into cDNA using a commercial kit (Vazyme, Nanjing, China). *ZmActin2* was used as the reference gene, and relative expression levels were determined using the 2^−ΔΔCt^ method, with transcript abundance at 0 h defined as the baseline for normalization [48].

### 4.4. Transcriptome Analysis

Maize root samples were collected for RNA extraction. Total RNA was isolated using a plant RNA extraction kit (Vazyme, Nanjing, China), and RNA quality was evaluated by NanoDrop analysis and 1% agarose gel electrophoresis. Qualified RNA samples were used for mRNA enrichment with Oligo(dT) magnetic beads, followed by mRNA fragmentation, cDNA synthesis, library preparation, and amplification. After quality assessment, the constructed libraries were sequenced on the Illumina NovaSeq platform. Raw reads were filtered to remove adapter-containing reads, reads with a high proportion of unknown bases, and low-quality reads. The quality of the resulting clean reads was evaluated using FastQC, and Q20 and Q30 values were calculated. High-quality clean reads were aligned to the *Zea mays* reference genome using HISAT2 v2.2.1. Gene-level read counts were obtained with featureCounts v1.5.0. Differential expression analysis was performed using DESeq2 based on the count matrix. Genes with an adjusted *p*-value (false discovery rate, FDR) < 0.05 and an absolute log_2_ fold change (|log_2_FC|) ≥ 1 were considered differentially expressed genes (DEGs). Gene expression levels were normalized as fragments per kilobase of transcript per million mapped reads (FPKM) values for expression pattern visualization and heatmap analysis, whereas normalized count data generated by DESeq2 were used for statistical testing of differential expression. GO functional classification and KEGG pathway enrichment analyses were performed using the OmicShare online analysis platform. For enrichment analysis, the RichFactor value was calculated as the ratio between the number of DEGs assigned to a specific pathway and the total number of genes annotated in that pathway. RichFactor was used to indicate the proportion of pathway-associated genes among DEGs and was considered an indicator of enrichment strength rather than a statistical criterion for defining significantly enriched pathways. Pathways were considered significantly enriched based on the adjusted *p*-value/FDR threshold (<0.05). Therefore, RichFactor values should be interpreted together with statistical significance rather than used independently for identifying enriched pathways.

### 4.5. Yeast One-Hybrid Assay

The pB42AD-ZmWRKY83 and pLacZi-ZmIAA7 constructs were co-transformed into EGY48 cells. Transformants were selected on SD/-Trp/-Ura medium. Positive colonies were spotted onto SD/-Trp/-Ura/Gal/Raf/X-Gal plates (80 μg/mL X-Gal). The plates were incubated at 30 °C in the dark, and blue coloration was observed to evaluate the interaction between ZmWRKY83 and the *ZmIAA7* promoter, as described previously [49].

### 4.6. DLR Assay

Recombinant plasmids carrying *ZmWRKY83* or *ZmIAA7* were introduced into GV3101. Bacterial cultures were collected and resuspended in infiltration buffer. The effector and reporter strains were mixed and infiltrated into tobacco leaves. After incubation in darkness for 72 h, leaf discs were collected for luciferase activity analysis. Firefly and Renilla luciferase activities were measured as previously described [50].

## 5. Conclusions

Taken together, our findings propose a model in which waterlogging stress suppresses ZmWRKY83 expression in maize roots, thereby relieving its repression of *ZmIAA7* and promoting auxin-mediated adaptive responses. Conversely, constitutive *ZmWRKY83* overexpression maintains *ZmIAA7* repression, disrupts auxin-related stress responses, and reduces waterlogging tolerance. This study identifies ZmWRKY83 as a negative WRKY regulator and reveals a ZmWRKY83–ZmIAA7 regulatory module involved in maize waterlogging responses, providing a potential genetic target for improving waterlogging tolerance. These findings expand our understanding of the functional diversity of WRKY transcription factors in stress adaptation and highlight that WRKY members may exert distinct or even opposite effects in regulating plant responses to the same environmental challenge. Moreover, the identification of the ZmWRKY83–ZmIAA7 module provides new insights into the integration of transcriptional regulation and hormone signaling during waterlogging stress, which may facilitate future genetic improvement of maize resilience under fluctuating environmental conditions.

## Data Availability

The original contributions presented in this study are included in the article. Further inquiries can be directed to the corresponding authors.

## Supplementary Materials

The following supporting information can be downloaded at https://www.mdpi.com/article/10.3390/plants15152358/s1, Table S1: The primers used in this study; Table S2. The sequencing quality statistics; Table S3. FPKM values of genes used for heatmap visualization; Figure S1. *ZmWRKY83* expression in *ZmWRKY83* overexpression line roots exposed to waterlogging stress. Error bars represent standard deviation (*n* = 3). Differences were analyzed using *t*-test; asterisks indicate significant differences, and ns denotes non-significant differences (*p* < 0.05); Figure S2. Cis-acting element analysis of the *ZmWRKY83* promoter. The 1500 bp upstream sequence of the ZmWRKY83 translation initiation site was analyzed for putative cis-acting regulatory elements. Different types of cis-elements are represented by colored boxes, including hormone-responsive elements, stress-responsive elements, light-responsive elements, and transcription factor-binding sites. The relative positions and distribution of identified cis-acting elements along the ZmWRKY83 promoter region are shown.

## References

[1] Erenstein O., Jaleta M., Sonder K., Mottaleb K., Prasanna B. (2022). Global maize production, consumption and trade: Trends and R&D implications. Food Secur..

[2] Behera S., Kar I., Yadav A., Sahu A. (2025). Exploring Waterlogging Challenges, Causes and Mitigating Strategies in Maize (*Zea mays* L.). J. Agron. Crop Sci..

[3] Liang K., Tang K., Fang T., Qiu F. (2020). Waterlogging tolerance in maize: Genetic and molecular basis. Mol. Breed..

[4] Striker G.G., Yamauchi T., Mollard F.P. (2025). Maize physiology under waterlogging: Shoot and root adaptive responses to mitigate low oxygen stress. J. Exp. Bot..

[5] Geng S., Lin Z., Xie S., Xiao J., Wang H., Zhao X., Zhou Y., Duan L. (2023). Ethylene enhanced waterlogging tolerance by changing root architecture and inducing aerenchyma formation in maize seedlings. J. Plant Physiol..

[6] Qi X., Li Q., Ma X., Qian C., Wang H., Ren N., Shen C., Huang S., Xu X., Xu Q. (2019). Waterlogging-induced adventitious root formation in cucumber is regulated by ethylene and auxin through reactive oxygen species signalling. Plant Cell Environ..

[7] Yan D., Gao Y., Zhang Y., Li D., Dirk L.M., Downie A.B., Zhao T. (2024). Raffinose catabolism enhances maize waterlogging tolerance by stimulating adventitious root growth and development. J. Exp. Bot..

[8] Liu Z., Yan J., Wang T., Chen W., Suo J., Yan J., Wu J. (2023). TgLCYB1 regulated by TgWRKY22 enhances the tolerance of Torreya grandis to waterlogging stress. Int. J. Biol. Macromol..

[9] Liu Z., Yan J., Chen J., Wang T., Li X., Wang S., Wang R., Liu Y., Yan J., Wu J. (2025). The TgIAA7-TgARF10-TgProDH1 module regulates waterlogging-mediated proline accumulation in the gymnosperm Torreya grandis. Plant Physiol..

[10] Yan J., Liu Z., Wang T., Wang R., Wang S., Liu X., Liu Y., Yan J., Wu J. (2026). TgRAV1–TgWRKY74–TgACS13 module fine-tunes ethylene biosynthesis to modulate waterlogging-induced root vitality in the gymnosperm Torreya grandis. J. Integr. Plant Biol..

[11] Wei X., Xu H., Rong W., Ye X., Zhang Z. (2019). Constitutive expression of a stabilized transcription factor group VII ethylene response factor enhances waterlogging tolerance in wheat without penalizing grain yield. Plant Cell Environ..

[12] Chen Y., Yin M., Sun L., Xu L., Yin Z. (2025). MYB gene family in Magnolia biondii: Identification and functional roles in waterlogging stress response. Plant Physiol. Biochem..

[13] Tian H., Fan G., Xiong X., Wang H., Zhang S., Geng G. (2024). Characterization and transformation of the *CabHLH18* gene from hot pepper to enhance waterlogging tolerance. Front. Plant Sci..

[14] Rushton P.J., Somssich I.E., Ringler P., Shen Q.J. (2010). WRKY transcription factors. Trends Plant Sci..

[15] Chen F., Hu Y., Vannozzi A., Wu K., Cai H., Qin Y., Mullis A., Lin Z., Zhang L. (2017). The WRKY transcription factor family in model plants and crops. Crit. Rev. Plant Sci..

[16] Eulgem T., Rushton P.J., Robatzek S., Somssich I.E. (2000). The WRKY superfamily of plant transcription factors. Trends Plant Sci..

[17] Chen L., Song Y., Li S., Zhang L., Zou C., Yu D. (2012). The role of WRKY transcription factors in plant abiotic stresses. Biochim. Biophys. Acta.

[18] Yang L., Fang S., Liu L., Zhao L., Chen W., Li X., Xu Z., Chen S., Wang H., Yu D. (2025). WRKY transcription factors: Hubs for regulating plant growth and stress responses. J. Integr. Plant Biol..

[19] Li X., Liu Z., Liu Y., Yan J., Suo J., Yu W., Wu J., Yan J. (2026). TgWRKY25-TgSBP1 module promotes cell expansion in Torreya grandis cones. Ind. Crop. Prod..

[20] Suo J., Liu Y., Yan J., Li Q., Chen W., Liu Z., Zhang Z., Hu Y., Yu W., Yan J. (2024). Sucrose promotes cone enlargement via the TgNGA1-TgWRKY47-TgEXPA2 module in Torreya grandis. New Phytol..

[21] Ding Z.J., Yan J.Y., Li G.X., Wu Z.C., Zhang S.Q., Zheng S.J. (2014). WRKY 41 controls Arabidopsis seed dormancy via direct regulation of ABI 3 transcript levels not downstream of ABA. Plant J..

[22] Miao Y., Laun T., Zimmermann P., Zentgraf U. (2004). Targets of the WRKY53 transcription factor and its role during leaf senescence in Arabidopsis. Plant Mol. Biol..

[23] Miao Y., Xu C., Zhang Y., Zhou H., Xu Q. (2025). OsMED25-OsWRKY78 Mediated Transcriptional Activation of OsGA20ox1 Positively Regulates Plant Height in Rice. Plant Cell Environ..

[24] Li T., Li B., Wang Y., Xu J., Li W., Chen Z.H., Mou W., Xue D. (2025). WRKY transcription factors in rice: Key regulators orchestrating development and stress resilience. Plant Cell Environ..

[25] Vanderauwera S., Vandenbroucke K., Inzé A., Van De Cotte B., Mühlenbock P., De Rycke R., Naouar N., Van Gaever T., Van Montagu M.C., Van Breusegem F. (2012). AtWRKY15 perturbation abolishes the mitochondrial stress response that steers osmotic stress tolerance in Arabidopsis. Proc. Natl. Acad. Sci. USA.

[26] Xing M.-y., Wang W.-q., Zhang C., Xi D.-j., Wang M.-c., Yin X.-r., Liu H., Liu X.-f. (2024). Identification and functional analyses of the transcription factors AcWRKY117 and AcWRKY29 involved in waterlogging response in kiwifruit plant. Sci. Hortic..

[27] Tang H., Bi H., Liu B., Lou S., Song Y., Tong S., Chen N., Jiang Y., Liu J., Liu H. (2021). WRKY33 interacts with WRKY12 protein to up-regulate RAP2. 2 during submergence induced hypoxia response in Arabidopsis thaliana. New Phytol..

[28] Gu L., Chen X., Hou Y., Wang H., Wang H., Zhu B., Du X. (2023). ZmWRKY70 activates the expression of hypoxic responsive genes in maize and enhances tolerance to submergence in Arabidopsis. Plant Physiol. Biochem..

[29] Li Q., Liu W., Zou H. (2026). ZmWRKY122 enhances waterlogging tolerance in maize via direct activation of *ZmPRX101*. Crop J..

[30] Korver R.A., Koevoets I.T., Testerink C. (2018). Out of shape during stress: A key role for auxin. Trends Plant Sci..

[31] Subbaiah C.C., Sachs M.M. (2003). Molecular and cellular adaptations of maize to flooding stress. Ann. Bot..

[32] Yan J., Liu Z., Wang T., Wang R., Wang S., Chen W., Suo J., Yan J., Wu J. (2024). TgLUT1 regulated by TgWRKY10 enhances the tolerance of Torreya grandis to drought stress. Plant Physiol. Biochem..

[33] Jiang J., Ma S., Ye N., Jiang M., Cao J., Zhang J. (2017). WRKY transcription factors in plant responses to stresses. J. Integr. Plant Biol..

[34] Tang Y., Guo J., Zhang T., Bai S., He K., Wang Z. (2021). Genome-wide analysis of WRKY gene family and the dynamic responses of key WRKY genes involved in Ostrinia furnacalis attack in Zea mays. Int. J. Mol. Sci..

[35] Hu W., Ren Q., Chen Y., Xu G., Qian Y. (2021). Genome-wide identification and analysis of WRKY gene family in maize provide insights into regulatory network in response to abiotic stresses. BMC Plant Biol..

[36] Daniel K., Hartman S. (2024). How plant roots respond to waterlogging. J. Exp. Bot..

[37] Yu F., Liang K., Fang T., Zhao H., Han X., Cai M., Qiu F. (2019). A group VII ethylene response factor gene, ZmEREB180, coordinates waterlogging tolerance in maize seedlings. Plant Biotechnol. J..

[38] Sun L., Ma L., He S., Hao F. (2018). AtrbohD functions downstream of ROP2 and positively regulates waterlogging response in Arabidopsis. Plant Signal. Behav..

[39] Pan J., Sohail H., Sharif R., Hu Q., Song J., Qi X., Chen X., Xu X. (2025). Cucumber JASMONATE ZIM-DOMAIN 8 interaction with transcription factor MYB6 impairs waterlogging-triggered adventitious rooting. Plant Physiol..

[40] Sharma A., Shahzad B., Rehman A., Bhardwaj R., Landi M., Zheng B. (2019). Response of phenylpropanoid pathway and the role of polyphenols in plants under abiotic stress. Molecules.

[41] Ran L., Li Y., Chen L., Mo T., Liu N., Xu S., Su Y., Wang C., Liang A., Zeng J. (2025). Specific expression of a fusion gene Lc-GhPAP1D results in colored cotton fibers with increased flavonoid and lignin synthesis, but impaired elongation and secondary cell wall deposition. J. Integr. Agric..

[42] Moustafa K., AbuQamar S., Jarrar M., Al-Rajab A.J., Trémouillaux-Guiller J. (2014). MAPK cascades and major abiotic stresses. Plant Cell Rep..

[43] Jalmi S.K., Sinha A.K. (2015). ROS mediated MAPK signaling in abiotic and biotic stress-striking similarities and differences. Front. Plant Sci..

[44] Lu H., Wang M., Li W., Chen Z., Li S., Yi Z., Zhang Y. (2023). Superior antioxidant capacity and Auxin Production promote seedling formation of Rice seeds under submergence stress. Agronomy.

[45] Shani E., Salehin M., Zhang Y., Sanchez S.E., Doherty C., Wang R., Mangado C.C., Song L., Tal I., Pisanty O. (2017). Plant stress tolerance requires auxin-sensitive Aux/IAA transcriptional repressors. Curr. Biol..

[46] Kumar A., Partap M., Warghat A.R. (2025). From growth to survival: Aux/IAA genes in plant development and stress management. Plant Sci..

[47] Xiang Y., Liu W., Niu Y., Li Q., Zhao C., Pan Y., Li G., Bian X., Miao Y., Zhang A. (2025). The maize GSK3-like kinase ZmSK1 negatively regulates drought tolerance by phosphorylating the transcription factor ZmCPP2. Plant Cell.

[48] Livak K.J., Schmittgen T.D. (2001). Analysis of relative gene expression data using real-time quantitative PCR and the 2−ΔΔCT method. Methods.

[49] Yan J., Zeng H., Chen W., Luo J., Kong C., Lou H., Wu J. (2023). New insights into the carotenoid biosynthesis in Torreya grandis kernels. Hortic. Plant J..

[50] Wang R., Wang T., Liu Y., Su J., Jin H., Wu J., Yan J. (2026). TgARAD1 regulated by TgERF8 enhances the tolerance of Torreya grandis to drought stress. Plant Sci..

