# Supplementary material for: ZmWRKY83 Negatively Regulates Waterlogging Stress in Maize
Source: Plants (Basel). 2026 Jul 31;15(15):2358. doi: 10.3390/plants15152358 (PMC13468163; doi:10.3390/plants15152358)
Supplement: Supplementary file 1 [file plants-15-02358-s001.zip › Supplemental Figure.pdf]

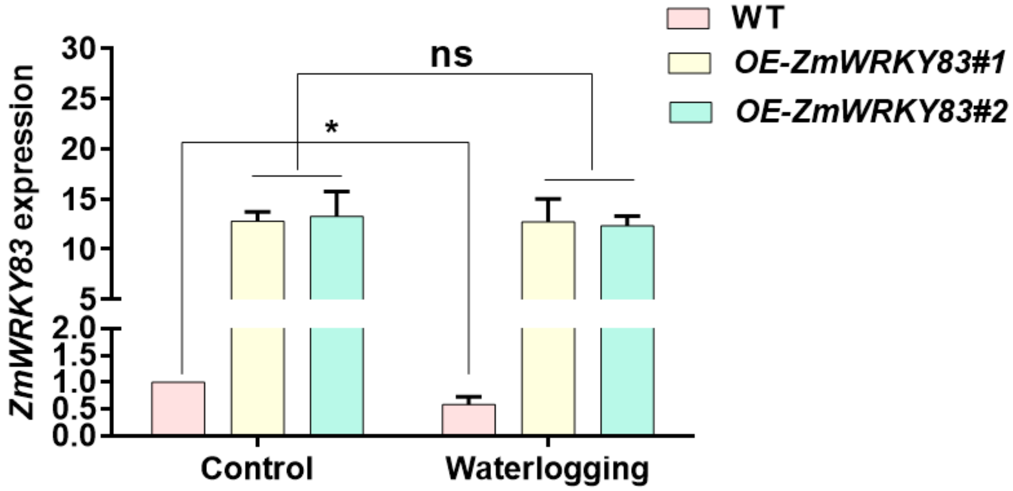

Figure S1. *ZmWRKY83* expression in *ZmWRKY83* overexpression lines roots exposed to waterlogging stress. Error bars represent standard deviation (n = 3). Differences were analyzed using t-test; asterisks indicate significant differences, and ns denotes non-significant differences ($P < 0.05$).

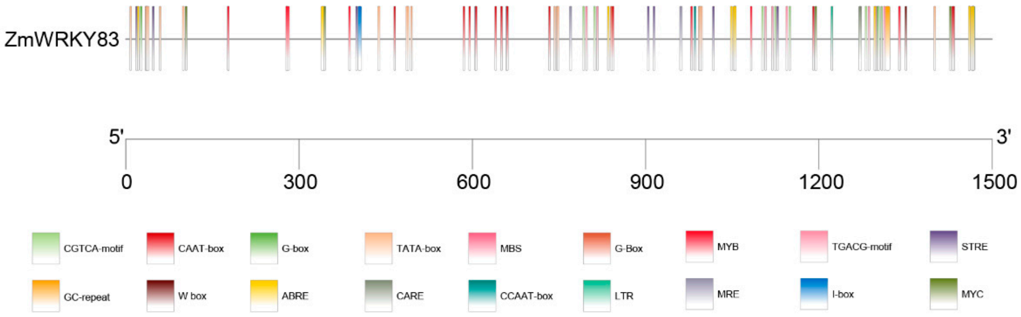

Figure S2. Cis-acting element analysis of the *ZmWRKY83* promoter. The 1,500 bp upstream sequence of the ZmWRKY83 translation initiation site was analyzed for putative cis-acting regulatory elements. Different types of cis-elements are represented by colored boxes, including hormone-responsive elements, stress-responsive elements, light-responsive elements, and transcription factor binding sites. The relative positions and distribution of identified cis-acting elements along the ZmWRKY83 promoter region are shown.
